# Transcription Blockage Leads to New Beginnings

**DOI:** 10.3390/biom5031600

**Published:** 2015-07-21

**Authors:** Leonardo C. Andrade-Lima, Artur Veloso, Mats Ljungman

**Affiliations:** 1 Department of Radiation Oncology and Translational Oncology Program, University of Michigan Medical School, Ann Arbor, MI 48109, USA; E-Mails: leolima11@gmail.com (L.C.A.-L.); abveloso@gmail.com (A.V.); 2 Department of Microbiology, Biomedical Sciences Institute, University of São Paulo, São Paulo 05508-000, Brazil; 3 Department of Computational Medicine and Bioinformatics, University of Michigan, Ann Arbor, MI 48109, USA; 4 Novartis Institutes of Biomedical Sciences, Cambridge, MA 02139, USA; 5 Department of Environmental Health Sciences, University of Michigan, Ann Arbor, MI 48109, USA

**Keywords:** DNA damage, DNA repair, RNA polymerase II, recovery of RNA synthesis

## Abstract

Environmental agents are constantly challenging cells by damaging DNA, leading to the blockage of transcription elongation. How do cells deal with transcription-blockage and how is transcription restarted after the blocking lesions are removed? Here we review the processes responsible for the removal of transcription-blocking lesions, as well as mechanisms of transcription restart. We also discuss recent data suggesting that blocked RNA polymerases may not resume transcription from the site of the lesion following its removal but, rather, are forced to start over from the beginning of genes.

## 1. Introduction

Transcription of DNA-encoded information involves RNA polymerases, which act like molecular motors pulling DNA through their active sites, generating complementary primary RNA molecules. In addition to the initiation step of transcription, RNA synthesis is regulated by the transition into the transcription elongation phase, the rate of elongation and transcription termination [1,2,3,4,5]. Following synthesis, the primary RNA molecules are spliced into mature forms and exported as RNA-protein complexes to ribosomes in the cytoplasm [6]. The fate of the mature RNA in the cytoplasm is regulated by miRNAs and RNA-binding proteins promoting mRNA degradation by cytoplasmic RNA exo-and endonucleases [7]. Damaged transcripts or transcripts containing un-spliced introns are targeted by a nuclear surveillance system consisting of the nuclear RNA exosome [8]. Together, RNA homeostasis is regulated and fine-tuned by complex transcriptional and post-transcriptional mechanisms.

Following exposure to DNA-damaging environmental agents, such as ultraviolet light (UV), the elongation process of transcription can be blocked [9,10,11]. Many chemotherapeutic agents, such as DNA topoisomerase I inhibitor camptothecin, can also affect the elongation phase of transcription [12,13,14]. Cells respond to elongation blockage by inducing cellular stress responses, involving both ATR and ATM, leading to activation of p53 and apoptosis [15,16,17,18,19,20]. Transcription-coupled nucleotide excision repair (TC-NER) is a specialized DNA repair system designated to remove lesions blocking transcription to increase resistance to the induction of apoptosis [15,17,21]. Apoptosis mediated by transcription blockage has been suggested to be a major contributing factor of the therapeutic activities of many chemotherapeutic agents [22]. In this review, we will focus on how DNA damage affects transcription elongation, how cells deal with transcription arrest, and how transcription recovery is accomplished.

## 2. Transcription Elongation—What is Blocking the Path?

**Figure 1:**
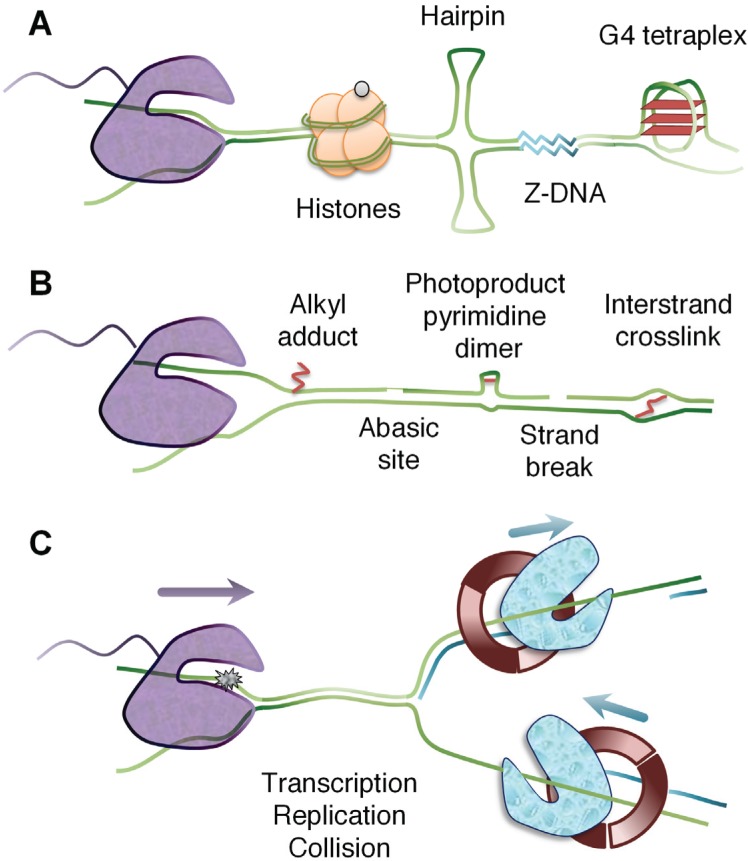
Blockage of transcription elongation. (**A**) DNA-binding proteins, such as histones, and non-B DNA structures, such as hairpins, Z-DNA, and G-quadruplexes. (**B**) DNA damage, including alkyl adducts induced by benzo[a]pyrene diol epoxide, N-nitroso; malondialdehyde or cyclopurines pyrimidine dimers induced by UV light; interstrand cross-links induced by platinum compounds (cisplatin, pyriplatin, phenanthriplatin); abasic sites and strand breaks induced as DNA repair intermediates. (**C**) Transcriptional arrest (abortion) due to convergent elongation of a replication fork.

Transcription elongation through chromatin in human cells has been estimated to occur at a variable speed of 0.5–5 kb/sec depending on the particular gene and the location within the gene [4,23,24,25,26]. Certain fixed gene features are known to slow down the rate of elongation, such as high GC content and a high density of exons [4,27,28]. Furthermore, non-B DNA conformations–such as hairpins, G-quadruplexes and Z-DNA–promote stalling of the RNA polymerase II complex. Using *in vitro* assays, it has been demonstrated that Z-DNA [29], repetitive DNA slip-outs [30], and G-quadruplexes in the non-transcribed strand [31] can block transcription elongation. Likewise, transcription of GC-rich stretches promotes RNA polymerase stalling, causing the formation of unusual stable RNA/DNA hybrid structures [32] (Figure 1A).

Recently, xeroderma pigmentosum factor B and D (XPB and XPD) helicases, which show dual roles in both transcription and nucleotide excision repair, were described to bind to and unwind G-quadruplexes [33]. Another helicase associated with RNA polymerase II is RECQL5, which has been implicated in preventing genomic instability by reducing replication fork collapse and double-strand break accumulation [34]. RECQL5 acts as an elongation factor, suppressing potential transcriptional stress by slowing down the elongation of RNA polymerase II [35]. Another mechanism of regulating the elongation rates of specific genes is by modifying histones. Genes showing fast elongation rates have been found to be associated with enhanced levels of di-methylation of lysine 79 of histone H3 (H3K79me2), while high CpG methylation in the bodies of genes correlates with slower elongation rates [4,25,26]. It is clear that RNA polymerases encounter numerous natural DNA structures and chromatin states that can impede its translocation and, if not dealt with appropriately, could lead to mutations or transcription-associated recombination [36,37,38,39].

### 2.1. DNA Damage on the Track

UV light is commonly used to model the biological consequences of DNA damage [40]. The first evidence that UV light inhibits transcription was reported already in 1962 [41]. Pyrimidine dimers induced by UV light distort the DNA double-helix, but this distortion does not fully explain transcription stalling. Rather, mis-incorporation of uridine into RNA across from the photoproduct is thought to cause the polymerase to stall [42]. Interstrand cross-links induced by cisplatin cause RNA polymerase II to stall before the lesion can enter the active site of the polymerase [43]. In contrast, monofunctional platinum compounds, including pyriplatin [44] and phenanthriplatin [45], induce bulky lesions that stall transcription complexes after addition of a CTP opposite the damage. Carcinogens, such as benzo[a]pyrene diol epoxide [46] and *N*-nitroso-derived adducts [47], have been reported to block transcription elongation, but the molecular mechanism of transcription blockage is not well understood. Oxidative stress can produce reactive aldehyde as a byproduct of lipid peroxidation and induce DNA adducts, such as malondialdehyde, [48] or cyclopurines [49], that cause transcription arrest (Figure 1B).

In addition to the direct inhibition of transcription by bulky lesions, transcription elongation can be inhibited indirectly by DNA damage repair intermediates. For example, oxidative DNA lesions, such as 8-oxo-G, do not, by themselves, constitute barriers to transcription, but when acted upon by DNA glycosylase OGG1 [50], or when bound by mismatch repair proteins [51], transcription is reduced. Base excision repair induces abasic sites as repair intermediates, which have been previously reported to represent strong blocks to RNA polymerase translocation [52]. These transcription-blocking abasic site intermediates are targeted by TC-NER [53]. Taken together, cells are challenged by endogenous and exogenous agents that interfere with RNA polymerase II progression and this has forced the development of several strategies for transcription stress avoidance, such as TC-NER and the removal of the stalled transcription complex through targeted ubiquitylation [54,55,56].

### 2.2. Transcription Meets Replication

The temporal ordering of replication firing of genomic regions during S-phase is strongly correlated to the transcriptional state of the chromatin. Early replicating regions are typically highly-transcribed while late replicating regions are mostly transcriptionally-inactive [57,58]. However, experiments using metabolic labeling of nascent DNA and RNA show that replication and transcription are spatially separated [58,59]. Thus, transcription is highly coordinated with replication in time and space as to not induce genomic instability. Transcriptional arrest by UV-induced lesions promotes apoptosis preferentially in S-phase, likely due to complications for the replication forks as they encounter blocked RNA polymerases [60]. Regardless of the presence of transcription-blocking DNA damage, very large active transcription units have been found to have a marked enhanced propensity for the induction of common fragile sites and copy number variations (CNVs) [61,62,63]. It has been proposed that replication origin firing has to occur outside the boundaries of these large transcription units and, thus, the DNA polymerases have to travel very long distances to complete these replication units. This may lead to transcription-dependent double-fork failure, resulting in unreplicated regions between them that would generate CNVs and fragile sites as cells progress into mitosis [63] (Figure 1C).

### 2.3. Why are Some Genes so Long?

Transcription elongation faces challenges from natural DNA sequences that have the propensity to form unusual DNA structures, complications with convergent replication forks, and from DNA-damaging agents inducing transcription-blocking lesions. In addition, transcription elongation is a very energy consuming process. With this in mind, it is astonishing to note how long some of the human genes have evolved to be. What may be some reasons for this? In cells, gene length converts into biological time where the expression of the mature RNAs of a set of specific co-induced transcripts can be separated in time with the smallest gene expected to finish first and the largest gene finishing last. For example, p53 induces a large number of genes simultaneously following DNA damage where some genes are short (CDKN1A is <10 kb) while other genes are very large (PAPPA is ~300 kb). The consequence of having the inducible genes being of different gene lengths is that the mature forms of RNA will be finished at different times bringing the corresponding mRNAs to the ribosomes in a sequential manner. Furthermore, large genes are more susceptible to gene inactivation by DNA-damaging agents [11,12,64] and thus, gene expression will shift in favor of small genes during times of DNA damage exposure as has been nicely described by McKay and co-workers [65].

## 3. Recovery of RNA Synthesis

### 3.1. TC-NER

Following UV light exposure, RNA polymerases stalled at sites of pyrimidine dimers act as DNA damage sensors, attracting nucleotide excision repair factors for TC-NER [21,66] and for the induction of an RPA and ATR-mediated stress response involving p53 [16,18]. TC-NER leads to faster repair of the transcribed strand of active genes compared to their non-transcribed strands or the genome, overall [21]. In *Saccharomyces cerevisiae*, two sub-pathways of TC-NER that depend on the functions of the RNA polymerase subunits Rpb9 and Rpb4 have been discovered [67]. Furthermore, the THO complex involved in mRNA biogenesis and mRNA export, and the transcription elongation complexes PAF and Ccr4-Not are required for TC-NER in yeast [68,69,70].

The mammalian TC-NER requires Cockayne’s syndrome factor B (CSB, an DNA ATPase), CSA (part of a E3-ubiquitin ligase complex), UV-stimulated scaffold protein A (UVSSA), and XPA-binding protein 2 (XAB2) to recruit the core NER components to the site of the blocked RNA polymerase II complexes [66,71,72]. Global genomic nucleotide excision repair (GG-NER) rely on damage surveillance by the XPC-hRAD23b and UV-DDB DNA damage recognition complexes. Incision and lesion removal results in a gap of about 30 nucleotides and this NER intermediate has been shown to trigger induction of H2AX phosphorylation and p53 accumulation [18,73]. This DNA damage response was observed in cells in G_1_-phase as well as in quiescent cells and supports the model that transcription blocks can act as triggers for the DNA damage response in a replication-independent manner [20,74,75].

### 3.2. Assessment of DNA Repair Genome-Wide

DNA deep-sequencing technologies have paved the way for the development of new tools to analyze the induction and repair of DNA damage in individual genes genome-wide. These approaches are based on cyclobutane pyrimidine dimer (CPD)-specific immunoprecipitation, followed by DNA microarray hybridization [76,77,78] or treatment with damage-specific endonuclease prior to deep sequencing [79]. Results obtained with these techniques suggest that CPD formation occurs with similar frequencies in genic and intergenic regions, with hotspots found adjacent to certain DNA repeat elements [76]. Excision repair sequencing (XR-seq) was developed to map sites of DNA repair, genome-wide, and is based on the isolation of excised damaged DNA via TFIIH immunoprecipitation followed by pyrimidine dimer immunoprecipitation [80]. Using XR-seq, a strong association between repair of the template strand and steady-state RNA expression levels was found, indicative of TC-NER. Furthermore, it was found that the repair near transcription start sites were more efficient than at the 3'-end of genes, which is similar to the result that we obtained using a technique called long qPCR that measures removal of UV lesions from specific sequences [11].

### 3.3. The Fate of Stalled RNA Polymerases

RNA polymerase II complexes stalled at UV-induced pyrimidine dimers give rise to footprints that are around 35 nucleotides long, indicating that the blocked RNA polymerases are effectively shielding the lesions [81]. It is therefore assumed that stalled RNA polymerases need to be displaced or forced to backtrack in order to allow access of the lesion to DNA repair factors. In bacteria, the product of a mfd (mutation frequency decline) gene couples transcription to DNA repair [82]. Mfd proteins bind to stalled RNA polymerases and promote their removal followed by the recruitment of NER factors to the exposed DNA lesions [83]. Surprisingly, Mfd-deficient cells are only mildly sensitive to UV light, suggesting that there may exist redundant mechanisms for removing stalled RNA polymerases in prokaryotes. Activated UvrD, with cooperation of the elongation factor NusA, have been described to promote the backtracking of the RNA polymerase II complex, allowing for the recruitment of the UvrAB complex to the site of damage [84,85]. While Mfd-dependent TC-NER results in polymerase removal, the UvrD-dependent TC-NER would potentially allow transcription to resume from the stalled site after lesion removal [86].

In eukaryotic cells, the TC-NER factor CSB has been found to possess ATPase activity that can wrap DNA [87]. However, it appears that it is not capable of either dissociating stalled RNA polymerases or act as a DNA helicase [88]. The CSB protein is associated with the elongating transcription machinery and this association becomes tighter after transcription stalling [89]. CSB is required as a coupling factor to attract histone acetyltransferase p300, nucleotide excision repair (NER) proteins, and CSA-DDB1 E3-ubiquitin ligase complexes to the stalled RNA polymerases [66]. UV-irradiation induces polyubiqutylation and degradation of the largest subunit of RNA polymerase II [90,91]. This process was initially thought to be CSB-dependent, since cells from Cockayne’s (CS) patients showed less proteasome-mediated degradation of RNA polymerase II [90,92]. It was later suggested that ubiquitylation of stalled RNA polymerase II is initiated by the ubiquitin ligase Nedd4 [93]. followed by sequentially-acting ubiquitin ligases [94]. In contrast to CS cells, Nedd4-depleted cells are not sensitive to UV light [93]. It is possible that DNA repair occurs without the need of the ubiquitylation and degradation of the stalled RNA polymerase [95]. In support of this view, CSB has been found to interact with XPG to promote TFIIH-dependent remodeling of stalled RNA polymerases to allow XPG to incise DNA lesions without the actual removal of the polymerase [96]. Interestingly, the mRNA 3'-end processing factor CstF has been shown to be recruited for the targeting of the stalled RNA polymerase by ubiquitin ligases, suggesting a link between mRNA processing and TC-NER [97].

**Figure 2:**
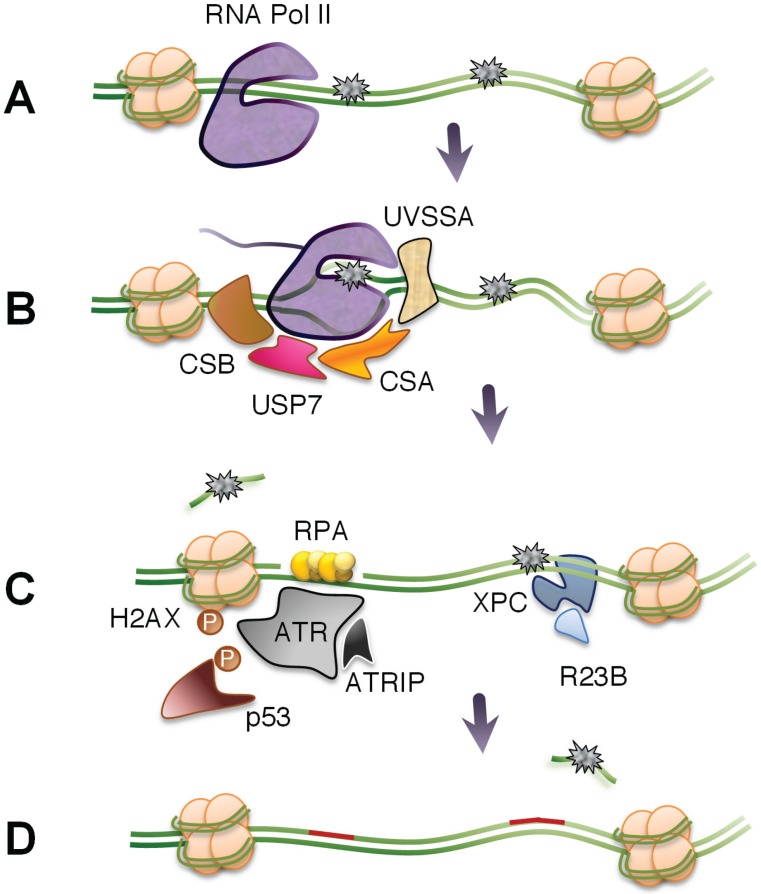
Removal of transcription-blocking DNA lesiosns. (**A**) Large genes are especially susceptible to gene inactivation by DNA-damaging agents. (**B**) Stalled RNA polymerase II promotes TC-NER by recruitment of CSB, CSA, UVSSA, and USP7 allowing for the remodeling of chromatin around the stalled RNA polymerase and the recruitment of core NER components. (**C**) Following removal of DNA damage, the gap formed may be extended by exonucleases and activate ATR kinase through RPA-binding to single-stranded DNA. Alternatively, blockage of transcription elongation may generate a single-stranded structure that binds RPA and activates ATR. The induction of the transcription stress response results in phosphorylation of ATR targets, including H2AX and p53. DNA lesions not associated with blocked RNA polymerases are substrates of GG-NER. (**D**) DNA repair is completed and the transcription stress response is diminished.

The UVSSA protein may also contribute to TC-NER by interacting with CSA proteins and the stalled RNA polymerase leading to the recruitment of ubiquitin protease USP7 that protects CSB from degradation [98,99,100] (Figure 2). The stabilization of CSB proteins may buy time for the remodeling of the stalled polymerase complex and for the recruitment of core NER factors. The UVSSA protein is also implicated in UV-specific ubiquitylation of RNA polymerase II but this ubiquitylation is not thought to lead to degradation of the polymerase [100]. It is possible that DNA lesions are removed before the degradation of the RNA polymerase by remodeling of the RNA polymerase resulting in displacement or backtracking. It would be reasonable to predict that, following RNA polymerase backtracking and photolesion removal, RNA synthesis would be able resume synthesis from the spot where it stopped. However, backtracking of mammalian RNA polymerase has only been observed using *in vitro* assays [101].

### 3.4. Factors Promoting the Recovery of RNA Synthesis

**Figure 3:**
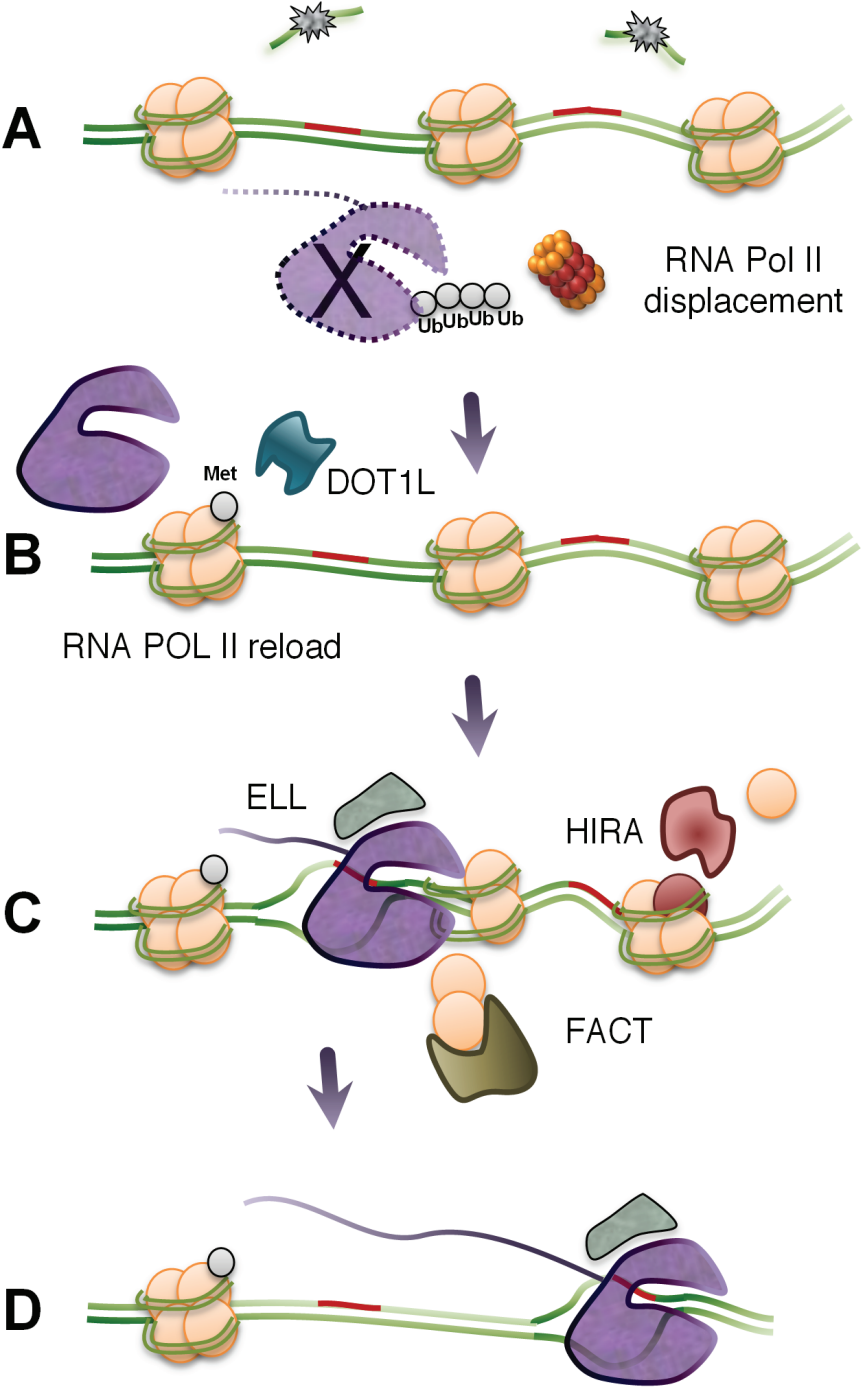
Transcription arrest leads to new beginnings. (**A**) As part of the DNA repair process, the RNA polymerase is displaced or degraded in a ubiquitin-dependent pathway. (**B**) Transcription starts anew from gene promoters. Specific histone modifications, including methylation of H3K79 by DOT1L, promote an open chromatin structure around promoters and control speed of transcription elongation. (**C**) Elongation factor ELL may serve as a docking site and contribute to transcriptional restart after repair. HIRA proteins are recruited to sites of repair where they promote the deposition of H3.3 histone variants into the chromatin. FACT proteins are also required for efficient restart and elongation of RNA polymerases as they stimulate H2A-H2B turnover. (**D**) Efficient transcription elongation through the repaired site.

How do cells resume transcription after removal of the blocking DNA lesion? A number of factors besides CSB and UVSSA proteins have been implicated to be required for RNA synthesis to resume following repair. One such factor is the RNA polymerase II elongation factor ELL, which interacts with the transcription factor TFIIH via the cyclin-dependent kinase 7 (Cdk7) [102]. Interestingly, ELL does not appear to participate in TC-NER, while CDK7 does. It has been suggested that ELL could serve as a docking site and promote transcriptional restart after repair (Figure 3). Other factors implicated in the recovery of RNA synthesis following UV-irradiation is transcription factor TFIIS and Crc4-Not [103,104]. Knockdown of TFIIS impairs transcription recovery and results in elevated levels of hyperphosphorylated RNA polymerase II. Interestingly, TC-NER activity do not seem to require TFIIS suggesting that TFIIS may play a post-repair function by aiding in the removal of the stalled phosphorylated form of RNA polymerase II.

Histone exchange and posttranslational modifications of the histones can affect both DNA repair and transcription. The lysine methyltransferase DOT1L methylates H3K79me2, which is a histone mark, is linked to the speed of transcription elongation [4,23,25,26]. The knockdown of DOT1L results in a strong impairment of transcription recovery following UV-irradiation, despite the normal removal of pyrimidine dimers [105]. It was suggested that by promoting an open chromatin structure around promoters, DOT1L stimulates the re-expression of genes following repair. It is possible that DOT1L may stimulate recovery of RNA synthesis both at the level of initiation and the level of elongation.

Chromatin remodeling has also been observed in the vicinity of induced UV lesions. Following UV-damage, Histone Regulator A (HIRA) proteins are recruited to sites of UV lesions where they promote the deposition of H3.3 histone variants into the chromatin [106]. Because H3.3 is found preferentially in the chromatin of transcriptional active genes, it was suggested that deposition of this histone variant into the chromatin in and around the UV lesions promotes RNA synthesis recovery by excluding specific transcription inhibitory factors. Histone chaperone Facilitates Chromatin Transcription (FACT) proteins are also required for efficient restart of RNA synthesis, as they accelerate H2A-H2B turnover in chromatin at sites of UV lesions [107]. Histone turnover may destabilize nucleosomes, which may facilitate backtracking or forward translocation of RNA polymerase II, but may also play a role in transcription recovery by diluting repressive marks associated with transcription silencing in damaged regions. Interestingly, both HIRA and FACT are recruited to regions of UV damage independently of DNA repair [106,107]. In contrast to recovery of RNA synthesis after UV-irradiation, HIRA and DOT1L are not needed for transcription restart after treatments with the transcriptional inhibitor 5,6-dichlorobenzimidazole 1-β-D-ribofuranoside (DRB), suggesting that their roles are specifically linked to transcription restart after DNA damage removal [108].

The tumor suppressor protein p53 has been implicated to play a role in promoting the recovery of RNA synthesis following UV-irradiation [109,110]. This role of p53 is dependent on functional TC-NER since reduced expression of p53 impaired recovery of RNA synthesis in normal and XP-C cells but not in TC-NER-deficient CS-B and XPA cells [111]. Whether the role of p53 in enhancing RNA synthesis recovery is due to regulation of TC-NER or involves a post-repair step is not presently known. Clearly, the function of p53 is distinct from its role in promoting expression of GG-NER by up-regulation of *XPC* and other genes [112,113]. While the role of p53 in regulating TC-NER is somewhat controversial [113,114], it has been shown that p53 acts as a chromatin accessibility factor promoting chromatin de-condensation [115]. Taken together, the chromatin-modifying factors discussed above may work cooperatively to orchestrate TC-NER and promote the recovery of RNA synthesis.

### 3.5. Resumption or Restart of Transcription?

We have developed Bru-seq to assess nascent RNA synthesis genome-wide [116,117]. This technique is based on the metabolic labeling of nascent RNA using bromouridine (Bru) and immunoprecipitation of the nascent RNA using anti-BrdU antibodies, followed by deep sequencing. We have used Bru-seq to explore the genome-wide effects of UV light on nascent transcription and we found that gene inactivation by UV-irradiation was proportional to the size of the gene with transcription elongation highly affected by, but without any apparent inhibition on, initiation of transcription [11]. Furthermore, RNA synthesis recovery following UV-irradiation occurred as a wave from the 5'-end of the gene with delayed recovery at the 3'-end of longer genes. The recovery at the 5'-end of long genes was more delayed in human XP-C fibroblasts, which are defective in global genomic NER (GG-NER) but proficient in TC-NER. In human CS-B fibroblasts, defective in TC-NER, the recovery was diminished at the 5'-end and abolished in the 3'-end of large genes. These results suggest that GG-NER promotes recovery at the 3'-end of long genes, while TC-NER is required for recovery throughout genes.

Our finding that nascent transcription recovers as a wave from the 5'-end of long genes following UV-irradiation could indicate that RNA polymerases blocked at DNA lesions must start over from the beginnings of genes rather than resume transcription from the sites of the removed lesions. In support for this model are findings using Bru-seq to explore the genome-wide transcriptional effects of the DNA-topoisomerase I inhibitor camptothecin [12]. Similar to UV light, camptothecin inhibits nascent transcription proportionally to gene size by blocking transcription elongation rather than inhibiting transcription initiation. While recovery of nascent RNA synthesis following UV-irradiation requires enzymatic repair to remove the blocking lesions, the inhibitory effects of camptothecin on transcription elongation is reversible and do not depend on enzymatic repair. Following washout of camptothecin, DNA topoisomerases rapidly finish their DNA isomerase reactions and depart from the DNA. This allows for the rapid recovery of nascent transcription. Similarly to the recovery after UV-irradiation, we found that the recovery of RNA synthesis following camptothecin removal occurred as a wave from the 5'-end of the genes with no recovery detected at the 3'-end of genes [12,118]. In contrast to the recovery of RNA synthesis following UV light, we found no role for CSB in the recovery of RNA synthesis following removal of camptothecin, indicating that the CSB protein is involved in TC-NER but not transcription restart. Interestingly, members of the spliceosome complex anchored to the phosphorylated C-terminal domain of elongating RNA polymerases, are actively removed from polymerases stalled at UV-induced lesions in a process partially dependent on the ATM kinase, which is activated by R-loops formed as a result of transcription stalling [20]. The disassembly of the spliceosome complex may indicate that the stalled RNA polymerase is destined for removal and/or destruction rather than preparing to resume transcription elongation.

Taken together, these findings strongly suggest that following transcription blockage, either at sites of a UV-induced lesion or trapped DNA topoisomerase I complexes, RNA polymerases are not permitted to resume transcription from the blocked site even after removal of the blockage but, rather, have to start transcription anew from the beginnings of genes. This scenario results in strong preference of nascent RNA synthesis recovery of small genes with a delay of expression of large genes that is proportional to its length.

## 4. Conclusions

DNA-damaging agents generated within cells or from exogenous sources are constantly challenging cells by damaging DNA and blocking transcription elongation. Inhibition of transcription is closely linked to the induction of apoptosis so there has been a strong evolutionary pressure to select for factors and processes that resolve these blockages and restore transcription [17,119]. However, if TC-NER and transcription recovery had been selected to be more efficient, there would have been a risk that the reset of the transcription stress response would occur too fast, prohibiting both thorough GG-NER and the induction of apoptosis, which would promote increased genomic instability [17,120].

TC-NER is a critical component of the RNA synthesis recovery process removing lesions that block transcription elongation. It has been hypothesized that RNA polymerases would be free to resume synthesis from the site where they were originally blocked after the lesions are removed. However, use of genome-wide next-generation techniques to monitor the effects of DNA damage on transcription initiation and elongation, as well as exploring how cells recover RNA synthesis following repair, provide us with a different picture. For both UV light and camptothecin treatment the data suggests that following removal of the blocking lesions, transcription restarts from the beginning of genes. This will lead to a delay in gene expression that is proportional to the length of the gene. Is this transcription delay important for the execution of the recovery process after UV light or camptothecin exposure? Has the transcription stress response exerted a selective pressure for gene length? Recent tools allowing for genome-wide interrogations of DNA damage and repair and for assessment of nascent RNA synthesis will undoubtedly lead to many new and exciting insights in this field of biology.
